# Typhoid Fever: Diagnosis and Treatment*Read before Tri-State Medical Society at Charlotte, N. C., January 20, 1899.

**Published:** 1899-02

**Authors:** Rolfe E. Hughes

**Affiliations:** Laurens, S. C.


### Southern Medical Record.

A MONTHLY JOURNAL OF MEDICINE AND SURGERY.

VOL. XXIX. ATLANTA, GA., FEBRUARY, 1899. NO. 2.

### Original Articles.

### TYPHOID FEVER: DIAGNOSIS AND TREATMENT.*

By ROLFE E. HUGHES, M.D.

Mr. Chairman, Gentlemen, Members of the Tri-State Society: If I could consistently think that your patience in listening to me would be rewarded by impressing you with any very new, valuable or radical suggestions, then indeed, with great calmness, I would exultingly treat at length the subject I have before me, that of Typhoid Fever, its Diagnosis and Treatment; but I realize being upon a well-beaten path, one that has been ably traveled by many eminent diagnosticians before, and certainly I would shrink from even treading cautiously upon a path so hard and worn, but for my enthusiasm inspired by the organization of this society, and from the fact that I am accompanied along the route by the most eminent men of three States. Most of the Virginia gentlemen present are personal friends, North Carolina's sons are our entertainers, and the Palmetto State, my adopted home, is well represented, so I at least will expect leniency of criticism; and should I lose my way, attribute it rather to a want of time for gathering full directions, and place me on the right route.

It is useless for me to add how honored I feel at being allowed to appear before a medical body of as high character as the Tri-State Society, and be permitted to participate in its scientific deliberations, 'Tis a happy moment for me and I shall ever remember gratefully and pleasantly these distinguished privileges, for aside from the real and scientific part, there is something inspiring and touching to see a congregation of medical men discussing the ills of mankind and the best possible means of combating them.

*Read before Tri-State Medical Society at Charlotte, N. C., January 20, 1899.

Man is heir to many troubles; our life-work is the study of these. 'Tis a serious responsibility and ofttimes a gloomy undertaking. Our life is spent on the darker side of human existence and misery, in the deepening shadows of which, as some writer has aptly said, "the God-like creations of the poet seem hideous masks." Still our duty is always the same—studying nature, vigilantly watching, cheerfully aiding. All through our professional lives, stern realities daily dawn upon us. Much of the sentiment, romance and tenderness of our real selves has been sacrificed in our search for truth.

Each day tells us more; the strides are rapid, newer fields are opened up, and now we are told of myriads of bacteria swarming in the air apparently the purest and lurking at so many places we least expect; that our cool, clear mountain spring is a favorite resort, and even that the Sacrament Cup and Holy Wine is not exempt. We further know many of our worst diseases are directly traceable to these organisms, each having his preferred mode of attack and selected soil. Probably one of the best known now is the bacillus of Eberth, which, by drinking-water, milk or what not, insidiously steals along the alimentary tract, and finding a suitable camping-ground at Peyer's patches, flourishes and multiplies to such an extent that he moves his habitat to neighboring tissues, and continuing to trespass, the integrity of the tissues is so upset that we have a patient, usually his age will be between fifteen and forty years (this of some diagnostic value); he has headache, insomnia, furred tongue, flagging energies, disturbed circulation, anorexia, epistaxis. He thinks he is "bilious;" we too often concur, order mercury and podophylin; patient departs, partakes of hog sausage, coarse beef, hard-boiled eggs, heavy bread, chicken salad, fruit cake and such poisons, when we are summoned again. Our diagnosis should have been made before, but the diagnosis of typhoid fever is not always easy; even in uncomplicated cases a careful and exhaustive physical examination of patient and investigation of surroundings is always necessary. All are familiar with the array of its symptoms so nicely arranged in text-books and few of us prepared for the serum tests, hence it is an individual study of each case and the intelligence of the practitioner that decides.

For instance, the characteristic temperature record of the so-called step-ladder temperature is in many cases a curiosity. The pulse is not necessarily characteristic and certainly not pathognomonic.

Enlargement of the spleen is of strong diagnostic value combined with others. It is usually palpable by the eighth day, and if there is not too much tympanites, this disappears as fever lessens. Septicæmia, malaria and miliary tuberculosis should here be excluded.

Rose-spots are important signs, and when present are typical, but I have never seen them in more than thirty per cent. of the cases; their appearance is usually on the ninth day. Gurgling in right iliac fossa must be looked for, but many intestine troubles have these, and all the diarrheas; so if accompanied by tenderness, and we can exclude appendicitis and pelvic abscesses, then we may expect typhoid.

The initial chill amounts to very little so far as as a diagnosis is concerned, and often as many as six chills may, at varying intervals, occur in unquestionable cases of typhoid. This is misleading, and in my section of South Carolina, where malaria abounds, often is the cause of confusion. Thus:

First, in typhoid we have the initial chill; second, chill at onset of relapse; third, result of antipyretics; fourth, at ushering in of complications, as pneumonia, pleurisy, acute otitis, suppuration in mesenteric veins, pyæmia, abscess of kidneys, perforation, etc.; fifth, during convalescence in bad cases; and sixth, when concurrent malaria exists—this is rare.

To recapitulate, I look for the following, and consider them accurate: Malaise, headache, chilliness, pain in back and limbs, tongue pale and indurated (in beginning); margins indented; it is put out slowly and retracted indifferently; there is confusion of ideas and mental torpor; tinnitus aurium, epistaxis, ascending pyrexia, photophobia, anorexia, rose-spots, gurgling and tenderness in right iliac fossa, constipation or diarrhea, and lastly vertigo. This I regard of great value. Patient on attempting to stand erect, trembles, and the nervous symptoms are pronounced. There a diagnosis is to be made from cerebro-spinal meningitis, for many cases of typhoid begin as typical meningitis. Fortunately, the latter trouble is rare, and other symptoms with surroundings usually settle doubts.

So much for the diagnosis; now how shall it be treated?

Within the last few years over eleven hundred remedies have been tried or suggested for typhoid fever. All sorts and kinds of food have been advocated; hydrotherapy and antipyretics for the temperature; intestinal antisepsis, etc. While special symptoms arising in the natural course of the disease have been experimentally dealt with by almost every resource of the pharmacopea there are few measures or means at the command of the physician which fulfill our wishes invariably, or even none which in all cases so far promises a specific; therefore he who adopts any one fad to the exclusion of all other efforts, be it in the line of hydrotherapy, antisepsis or what not, fails in his duty to his patient, his profession or himself. It has been said, "The best treatment is a good physician." One knowing the natural course of the disease and able therefore to intelligently anticipate its various complications and phases; knowing these, how can he have any radical fixed schedule which can be best for all cases and their inconstances?

He should be watchful and conservative, backed by the judgment to adopt such measures as his good sense will in individual cases dictate. It may be Woodbridge or Brand, in whole or in part, or probably a happier medium by the combined antiseptic and hydrotherapy methods; judgment must be exercised in mode or manner of each. Unquestionably hydrotherapy, variously modified, is for its indications the best treatment known, always to be used in hospitals and in private practice when possible. The treatment summarized embraces:

1. General management and nursing.
2. Diet.
3. Treatment of the temperature.
4. Antiseptic medication.
5. Treatment of special symptoms.
6. The convalesence.

*General Management.*—A typhoid patient should be in a cool, well-ventilated apartment, confined to the bed from the onset, and remaining until convalescence is well established. The woven-wire bed with soft hair mattress is best. A rubber cloth should be under the sheet, a good nurse in charge, and the physician should at each visit write out specific instructions.

*Diet.*—Foods easiest digested and obviously those leaving behind the smallest amount of residue. Milk heads the list, and ordinarily about three pints for an adult in twenty-four hours. 'Tis often advisable to dilute this with water, lime water or even aerated waters. It should be given at regular intervals and care exercised, for too much leaves masses of curds and thus proves irritating. The usual broths, chicken or mutton, come next, and many of the beef juices are good.

Water should be given in abundance and pleasantly cool. My experience justifies making this of paramount importance and I wish I could go into the merits.

*Treatment of the Temperature.*—Bathing or sponging and not the use of coal tar antipyretics. Sponging with cool water is the preferred practice with the most successful physicians of my section. Dr. J. P. Simpson, of Laurens, S. C., who deservedly enjoys a great reputation and has had extensive experience with typhoid fever, has, to my certain knowledge, had amazing success in the last two years. Dr. Simpson's method of administering the bath is to place a wire cot by bed, upon this lay a piece of oilcloth and cut opening in center for water to escape into vessel below, weight of patient causing the cloth to dip, thus throwing water to center; patient stripped, is sponged with water, temperature 70. In some instances it is poured over, always using it in temperature of 102½ or 103 and repeating as necessary to control this temperature; friction is used while administering and to point of bringing glow; cold pack to head.

*Antiseptic Medication.*—The efforts to settle upon an agent destructive to the typhoid bacilli or the toxic agent they produce, so far is a failure, and we adopt George's plan of the chlorine waters; it has not been satisfactory, but certainly does no harm, therefore is a pious fraud. The intentions are good. For my own part, salol is the remedy when no cardiac complications exist.

*Treatment of Special Symptoms.*—For tympanitis and abdominal pain, turpentine stupes; diarrhea, if severe, starch and laudanum enemas or subgallate of bismuth; no opium, even in form of Dovers powders. Constipation, if demanding notice, is best met by Hunyadi water.

As to hemmorrhage and peritonitis, the two grave complications, all are familiar with and I suppose all treat them alike.

For the nervousness, hydrotherapy again comes, and cold water to head; after the sponging and application of cold water cap, the result is usually so satisfactory as to admit of no strictly medicinal sedative.

Brandy and strychnia head the list for progressive heart failure; digitalis is not good.

Convalesence requires even as much care and watching as the acute and active stage of the disease. Over-eating and too much exercise are usually the causes of relapse. The other minor details fall in line too naturally to tax your patience here, so I relieve you.

Laurens, S. C., January 17, 1899.

---

REGARDING TUBAL MENSTRUATION.—By Dr. H. Thompson (*Centbl. f. Gyn.*, 1898, No. 45).—The question of a menstrual process in the tubes similar to that in the uterus is still disputed. There are but three cases on record free from objection, and the author adds a fourth.

In a case of ruptured abscess, a tubo-abdominal fistula was left, remaining eight months. Through this opening a bloody discharge occurred simultaneously with the uterine discharge, and ceased with the termination of the regular menstrual epoch. The curious combination was observed a number of times, without other coexisting symptoms, until a few silk ligatures, with which the pedicle had been ligated, had passed, and aft[illegible] which the fistula rapidly closed.

Ma[illegible] and Leopold showed, through anatomical researches, that during menstruation the tubal mucosa underwent changes similar to those occurring in the uterine mucosa, although to a milder degree.

